# Financial assessment and diagnosis of abnormal data of listed companies in public health and wellness industry

**DOI:** 10.3389/fpubh.2024.1508389

**Published:** 2024-12-20

**Authors:** Xiao Liu, Yanhong Li, Shengnan Zhang

**Affiliations:** College of Finance and Economics, Wan Jiang University of Technology, Maanshan, China

**Keywords:** public health, wellness industry, financial risk assessment, abnormal data diagnosis, health and safety

## Abstract

In this study, the present status of the public health and wellness sector in China is examined. The main objective is to evaluate the financial risk among publicly listed companies in the sector. Despite the significant expansion of the industry, research in this domain remains limited. Moreover, it frequently lacks depth, especially when it comes to financial studies designed especially for publicly traded companies. Current models for assessing corporate financial risk are frequently applied in diverse industries without necessary adjustments. Therefore, this study examines the financial performance and operational health of five publicly listed anonymized companies between 2020 and 2023. Using an efficient methodology that includes reliable data collection and quality assurance, we analyze key performance indicators such as annual revenues, liquidity ratios, and debt-to-equity ratios to assess financial stability and growth potential. Our findings reveal varied revenue trends shaped by external market shocks such as the COVID-19 pandemic. These trends highlight both the resilience of the sector and its vulnerabilities. To evaluate potential financial risks and outliers, the abnormal revenue patterns are also identified using Z-score. Benchmarking is also conducted using industry averages which provides a clearer understanding of competitive positioning. Several suggestions are made in light of the analysis to manage debt, increase liquidity, and leverage digital transformation for long-term growth. Several future directions are also presented to continue research in the domain. Overall, this study offers valuable information for stakeholders navigating the complexities of the public health and wellness industry in China.

## 1 Introduction

The initiative “Community of Human Health and Wellness” was first proposed on 21 March 2020 by President Xi Jinping. Proposed during a telephone meeting with French President Macron, this global public policy initiative has received a great deal of attention from various countries and scholars around the world (1). Later, in 2022, “promoting world peace and development, and promoting the building of a community of human destiny” was explicitly proposed at the 20th Party Congress, which also noted that “the human health community and the community of human destiny are in the same vein” (2). The introduction of this initiative has catalyzed the growth of the public health sector, with the enhancement of public health emerging as a key developmental objective that scholars are increasingly focusing on. Of course, the prevention and control of disease, regulation of medical supply, protection of human health and rehabilitation all belong to the scope of public health and are key components of the people's health service system. Under such a general environment, the development of listed enterprises in the public health industry is strongly supported by the state. Therefore, the financial evaluation and abnormal data diagnosis of these listed enterprises are particularly important, and a large number of scholars have also studied the financial risk evaluation of other industries. Among them, in the current social background of the developed Internet, Fang Dan believes that we should try to combine the background of this era, the financial risk prevention and control system and the Internet to create an advantageous Internet financial prevention and control technology, and then to achieve intelligent control and management of the enterprise's financial risk, in order to enhance the financial risk prevention and control ability of listed enterprises (3). Guoliang et al. conducted a detailed study of different financial data analysis methods, and chose to quantify some of the intricate relationships that exist in listed enterprises through principal component analysis, which means that the financial risks of listed companies in the real estate industry were studied in data, and then the financial risks of listed companies and anomalies could be monitored, which can be used to carry out operable management of the financial risks of listed enterprises (4). Rodgers et al. through the Logistic model to assess the financial risk of listed companies, the introduction of cash flow interest rate data to distinguish whether the listed company is profitable, which is more conducive to the listed company's knowledge of their own development, can be healthier and long-term development in the competitive environment (5).

Given the current limited research on financial risk assessment among listed companies across various sectors, there is a particular scarcity in the public health and wellness industry. This study aims to fill this gap. It conducts a comparative analysis of domestic and international studies on financial risk assessment and the diagnosis of abnormal data. The research clarifies and examines the evolution of financial risks in these companies. It also evaluates the methodologies employed in their analysis. The key contributions of this paper are summarized as follows:

1. This study provides a detailed evaluation of revenue trends and operational health in publicly listed companies within the public health and wellness sector.
2. Initially, this study examines critical financial health ratios, including liquidity and debt-to-equity ratios. This offers insights into the companies' liquidity, wealth, and overall financial risk. Additionally, it benchmarks these companies against industry averages to highlight the competitive positioning and identifying areas for potential improvement.
3. Thereafter, this study analyzes revenue growth in relation to economic disruptions and health crises. By employing statistical tools like Z-scores, it identifies abnormal revenue patterns, ensuring data integrity and accuracy in financial assessments.
4. Finally, the study provides various recommendations for managing debt, enhancing liquidity, and utilizing digital transformation to support long-term growth in the sector.

## 2 Literature review on financial risk assessment

### 2.1 Foreign research progress

Dating back to the early 1930s, the genesis of financial risk literature began to integrate quantitative analyses with qualitative inquiries. Scholars like Jordan et al. initiated a somewhat explanatory account of financial risk, defining it as changes in funding due to uncertainty in financial decisions, which lead to alterations in debt profiles (6). Beaver further refined the definition by equating insolvency with financial risk (7). During this period, the concept of financial risk was widely introduced, sparking a surge in scholarly interest and rapid development of related research after the 1970s. In 2002, Marjorie advanced the financial risk assessment model by combining liabilities and profit-related indicators, along with an analysis of the company's total amount, to achieve a more precise financial risk assessment model (8). Consequently, an increasing number of scholars have come to recognize that the accuracy of financial risk assessment models is closely related to the key indicators of the industry under scrutiny. In the context of real estate listed companies, Tian et al. have emphasized the importance of selecting indicators relevant to the industry for comprehensive analysis. Factors such as external financing capacity and cash flow are considered, followed by a combination of qualitative and quantitative analysis methods to more accurately monitor and predict the financial risks of listed companies in this sector (9). This approach underscores the significance of industry-specific indicators in the financial risk assessment process. The evolution of financial risk assessment has been marked by a shift from general to more industry-focused models, reflecting a deeper understanding of the nuances within different sectors. As the field continues to evolve, the integration of industry-specific indicators and advanced analytical techniques will likely play a crucial role in enhancing the accuracy and relevance of financial risk assessments.

### 2.2 Progress of domestic research

Compared to the early foreign research on financial risk dating back to 1930, China's exploration of the topic commenced somewhat later. In 1994, Xiang provided a more nuanced classification of financial risk, offering detailed descriptions for each category (10). This work marked a significant step forward, as it prompted a surge in related studies in China, encompassing the causes of financial risk and the modeling of financial risk assessment. Zhang and Gao conducted a comprehensive analysis of the causes of financial risk, attributing the financial issues of listed companies to the combined impact of internal and external factors (11). Tang and Jia, on the other hand, focused primarily on internal causes, examining the risks inherent in every aspect of enterprise development (12). Similarly, Zhang and Yu analyzed the internal factors leading to financial risk, highlighting the uncertainties in internal financial management and the predominant role of human factors (13). This perspective closely aligns with Wu's analysis, which also pointed to the inadequacy of financial personnel's capabilities or their lack of sensitivity to financial risks (14). Conversely, Liu and Wu, in their research on domestic financial risk assessment, discovered that big data could be integrated with the financial risk assessment index system, leveraging the power of computers to construct a more precise model (15). Zheng, in his research on domestic financial risk assessment, not only extracted the principal component scores of enterprise financial risk through principal component analysis but also proposed a relevant financial risk assessment model (16). Additionally, a multitude of scholars have employed the Analytic Hierarchy Process (AHP) to prioritize enterprise financial indicators, thereby establishing a comprehensive financial risk assessment system tailored to specific industries. These efforts underscore the importance of industry-specific indicators in enhancing the accuracy and applicability of financial risk assessment models.

## 3 Current situation of public health industry

**Figure 1:**
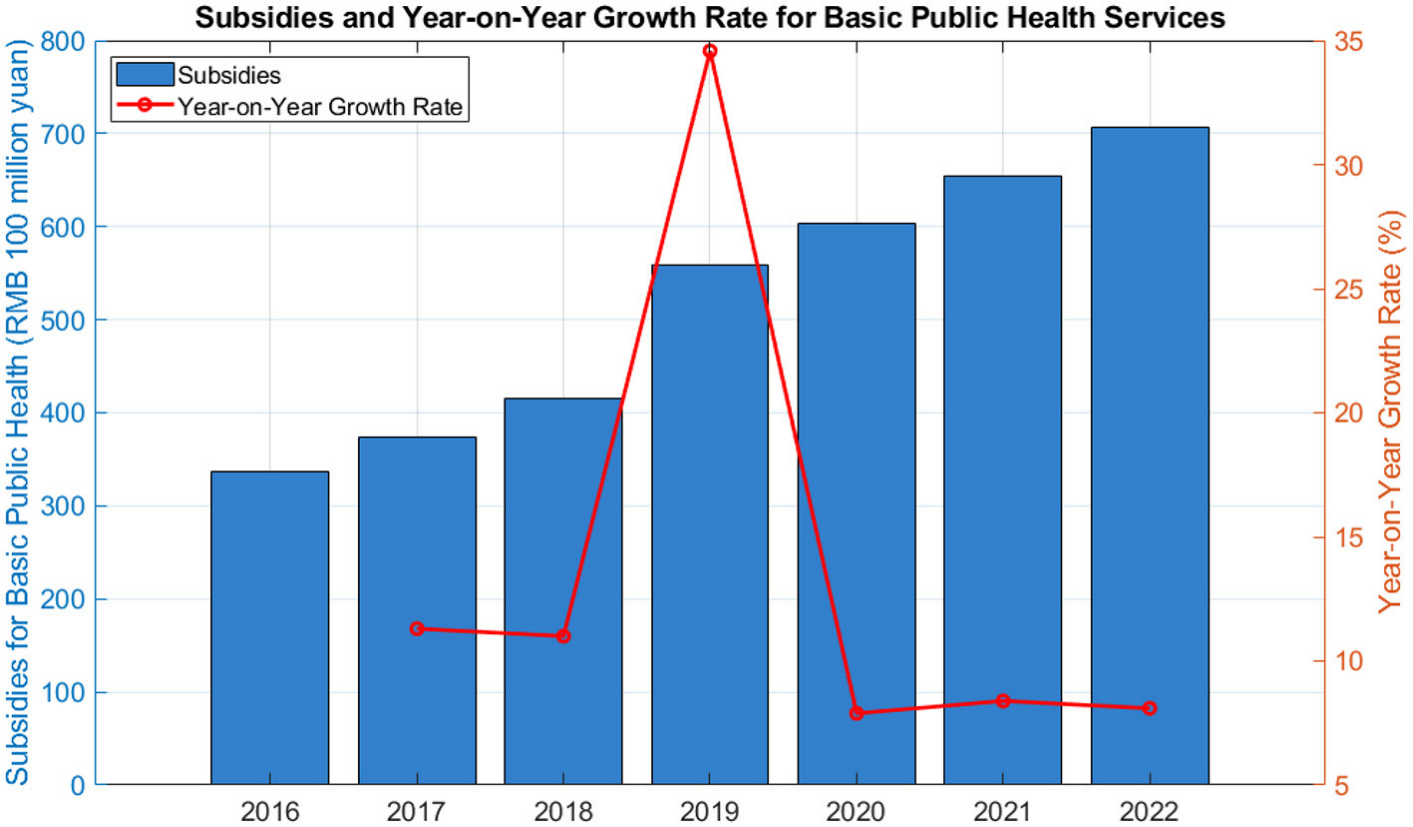
Government subsidies for public health services.

With China's robust policy support for the public health and wellness industry, the sector is poised for broader development and opportunities. This industry encompasses a range of areas, including health services, healthcare, and rehabilitation, among others. Notably, the integration of high and new technology in health monitoring and management is increasing public reliance on related products, which in turn is significantly contributing to the industry's growth. Figure 1 shows the state's investment in public health service subsidies indicates a growing emphasis on the industry. From 2016 to 2022, the funding for basic public health service subsidies has been on the rise. In particular, the subsidy in 2019 saw a notable increase of 55.92 billion yuan compared to the previous year, according to the Ministry of Finance. This trend underscores China's increasing focus on the public health and wellness industry, suggesting that listed companies in this field will enjoy expanding development opportunities. Furthermore, the National Health Commission has reported an increase in per capita government subsidies for basic public health services, raising it to 79 yuan (around $12.2) in 2021, which is five yuan more than the previous year. These additional funds are directed towards enhancing public health services, especially for vulnerable groups such as seniors and children, and for epidemic prevention and control at the community level. The government's commitment to advancing digital health records and improving services for key populations, like those with chronic diseases and individuals aged 65 and above, also signals a continued investment in the public health and wellness industry. These measures are expected to further stimulate the industry's prosperity and provide a solid foundation for listed companies to flourish in the coming years.

**Figure 2:**
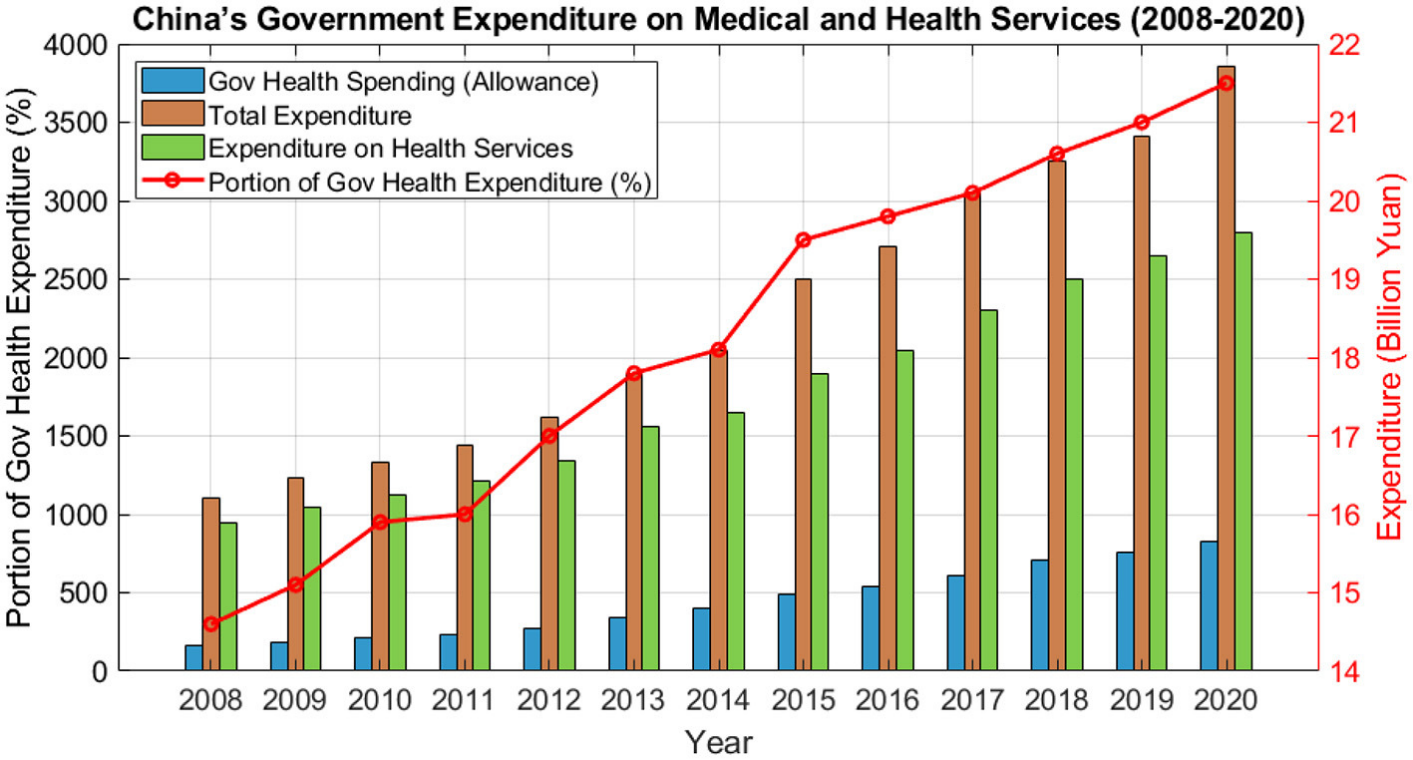
China's government expenditure on medical and health services, 2008–2020 (unit: billion yuan).

Based on the annual increase in national subsidies for public health services, an analysis of China's government expenditure on medical and health services (Figure 2) reveals that in 2020, China's government expenditure on medical and health services will be 2.19 trillion yuan, which will account for 8.41% of the government's financial expenditure (Source: China Health Statistical Yearbook).

The state of the investment and expenditure of the public health service subsidy fund in medical and health services not only reflects the importance of the public health and wellness industry in the national development plan, but it is more related to the health and safety of our people and property income. Among them, in China's residents of the fund of financial research and forecasting to get Figure 2. It is known that China's financial mainly for individual contributions to a part of the government subsidies, but also to the residents of the basic pension subsidies, and finally to the residents of personal account fund of the annual income and expenditure of the shortfall in the subsidies. From the Figure 2, it can be seen that the financial investment in urban and rural residence insurance is gradually increasing, and it is predicted that by 2100 it can exceed 20%. At the same time, the ratio of fiscal expenditure on urban and rural residence insurance to GDP is 1.27 per cent in 2020, and is forecast to exceed 2 per cent in 2050, with an increasing trend year by year.

**Figure 3:**
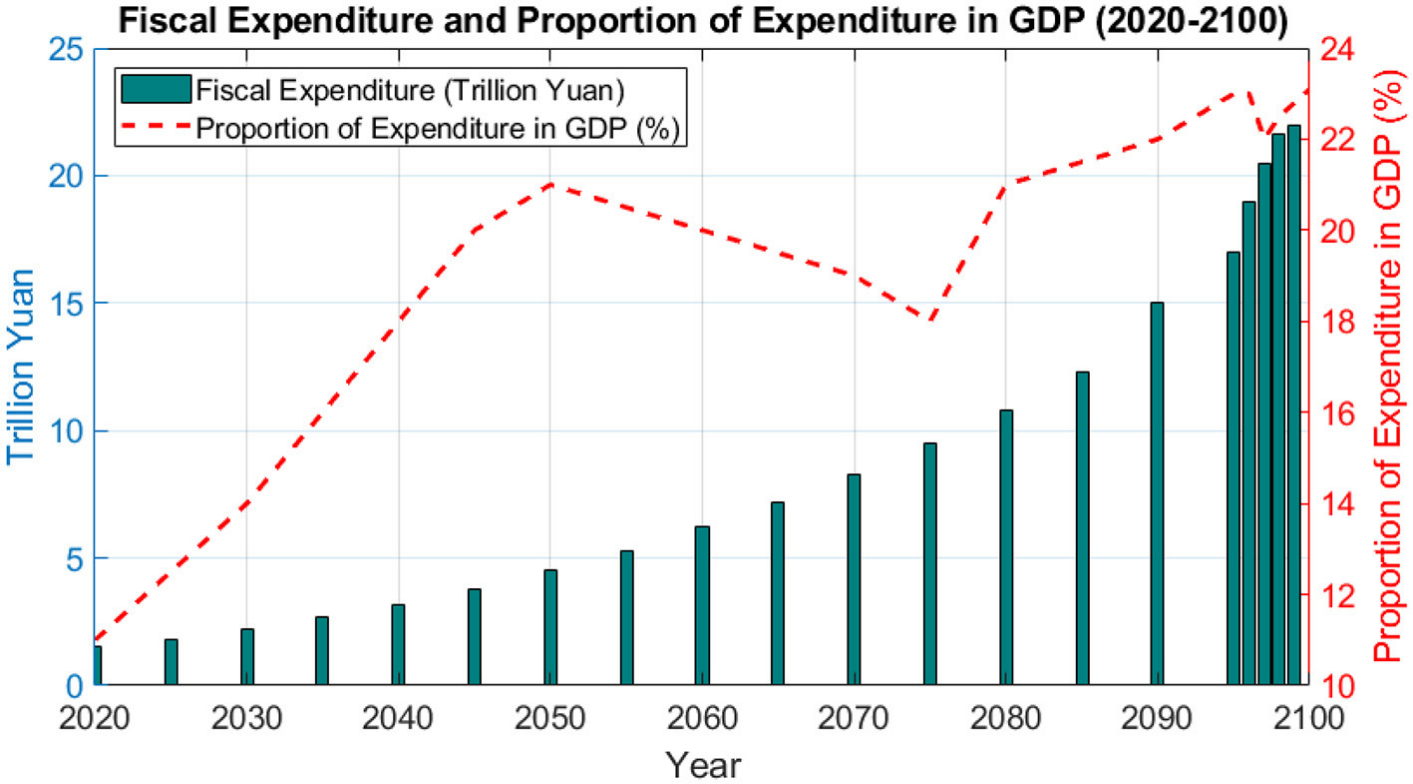
Fiscal expenditure and GDP share of urban and rural home insurance.

**Figure 4:**
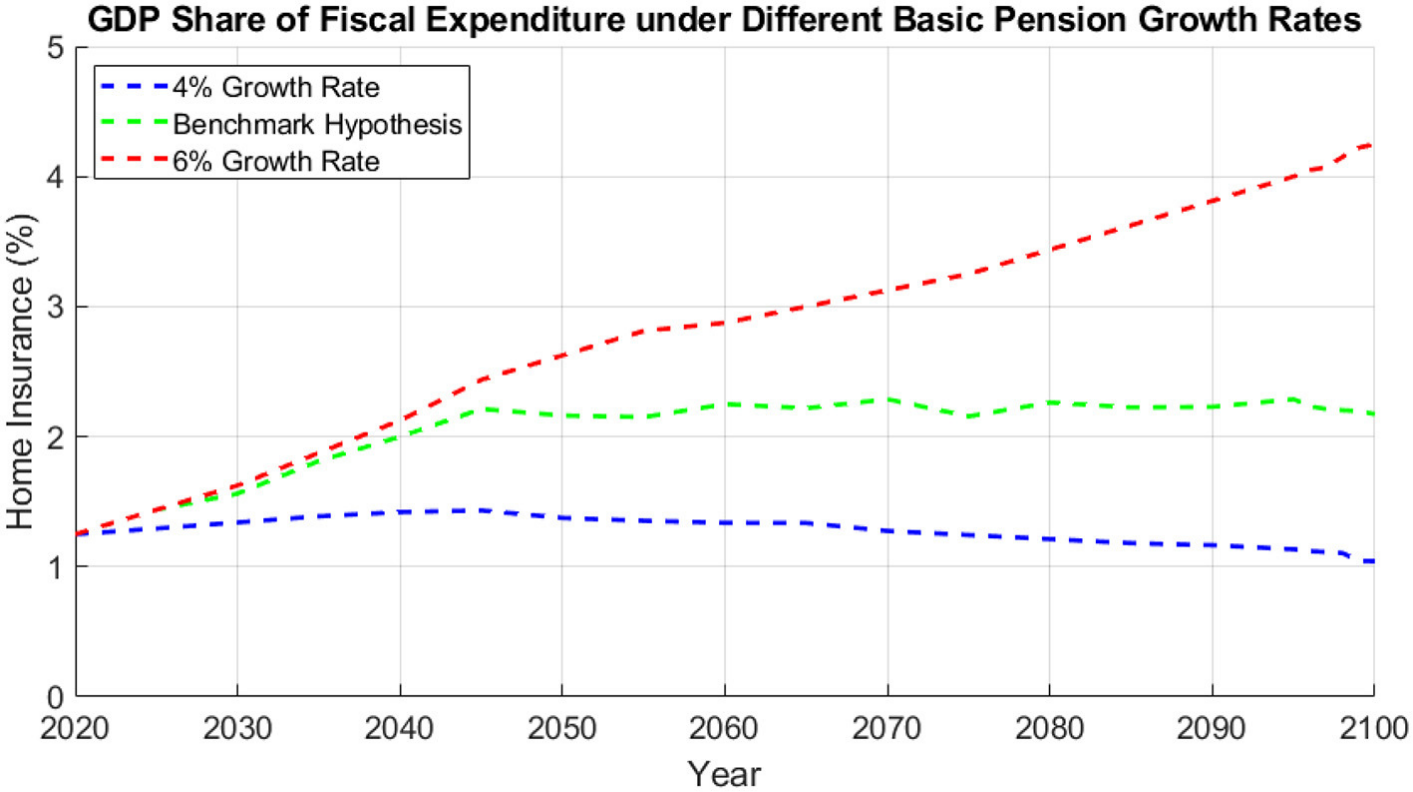
GDP share of fiscal expenditure on urban and rural home insurance under different basic pension growth rates.

After the previous analysis of the fiscal expenditure and GDP share of urban and rural home insurance, further research on the fiscal expenditure and GDP share of urban and rural home insurance under different growth rates of the basic pension in China is shown in Figure 3. The changes in the growth rate of the basic pension are not very much correlated with the income and expenditure of the personal accounts and liabilities of the residents, which is due to the fact that the basic pension is paid through the state treasury. As the basic pension continues to grow, it makes the expenditure of the state treasury higher and higher, which will ultimately reduce the support of the state treasury for urban and rural residence insurance. Figure 4 shows that the different growth rates of the basic pension start with no difference in 2020 and produce large differences by 2100. Among them, when the growth rate of the basic pension is 4%, the GDP share of financial expenditure on urban and rural residence insurance reaches the highest in 2055, and then continues to decrease. While when the growth rate of the basic pension is 6%, the GDP share of financial expenditure on urban and rural residence insurance enters into a period of slow increase in 2055, and then it will be rapidly increased, and even exceeds 4% in 2100. This means that the growth rate of the basic pension will largely have the opposite effect on the expenditure of the rural and urban insurance finances.

Therefore, China's public health and wellness industry is not only reflected in the government's investment in public health service subsidies, but also in the government's medical and health service expenditures, which involves financial expenditures from urban and rural residence insurance and the GDP ratio and the GDP ratio of urban and rural residence insurance under the growth rate of different basic pensions, the support of the national policy and residents have money in their hands, which has become a driving force for the healthy and long-term development of the public health and wellness industry. The driving force for the healthy and long-lasting development of listed companies.

## 4 Dataset collection and processing

**Figure 5:**
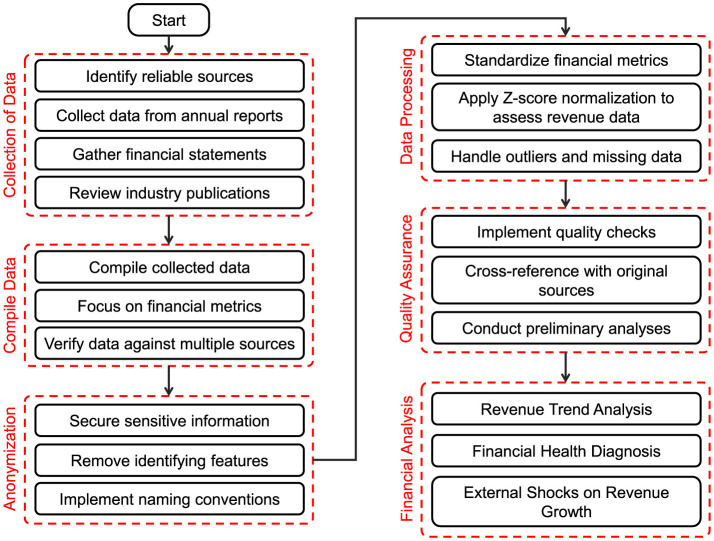
Diagrammatic flow of the financial assessment and diagnosis of abnormal data of listed companies in public health and wellness industry.

In this study, we focus on analyzing data from five well-known publicly listed companies in the public health and wellness industry. Figure 5 shows the diagrammatic flow of the financial assessment and diagnosis of abnormal data from listed companies in the public health and wellness industry.

To ensure confidentiality and protect sensitive information, we have anonymized the companies by assigning them designations as C1 through C5. This approach allows us to present our findings without compromising any proprietary or sensitive data related to the companies involved.

To compute financial ratios and conduct a thorough analysis, it is essential to utilize a combination of theoretical foundations and practical insights from financial management literature. According to Brigham et al. (17), financial ratios serve as critical tools to assess company performance and financial health. These ratios allow stakeholders to make informed decisions based on quantitative metrics. They facilitate comparisons across time periods and with industry benchmarks. This provides a clearer picture of operational efficiency, profitability, liquidity, and solvency.

Moreover, Koller et al. (18) emphasize the importance of valuation techniques in conjunction with financial ratios. This combination is necessary to derive a comprehensive understanding of a company's market value and underlying economic performance. By integrating ratio analysis with robust valuation methodologies, analysts can identify trends and evaluate managerial effectiveness. This integration supports strategic recommendations that enhance corporate value and stakeholder wealth.

The data collection process involved the following key steps:

1. **Data sources**: We sourced financial and operational data from reliable and publicly available databases, including annual reports, financial statements, and industry publications. These sources provided comprehensive insights into each company's performance over the specified period from 2020 to 2023.
2. **Data compilation**: The collected data was meticulously compiled to ensure its accuracy and reliability. We focused on essential financial metrics, including annual revenues, liquidity ratios (current and quick ratios), debt-to-equity ratios, and revenue growth percentages. Each data point was verified against multiple sources to mitigate errors and ensure consistency.
3. **Anonymization**: Drawing on insights from Gadotti et al. (19), Xu and Zhang (20), and El Emam and Dankar (21), a careful anonymization process was used to ensure privacy and reduce potential bias. All sensitive data was protected, and identifying information was removed or modified. Gadotti et al. highlight the challenge of balancing data usability with privacy in anonymization practices. Xu and Zhang discuss how anonymization can affect statistical results, especially in detecting disparities, emphasizing the need for careful application. El Emam and Dankar's work on k-anonymity informed our approach by promoting techniques that protect individual identities while preserving the usefulness of the data for analysis.
4. **Data processing**: After compiling the data, processing steps were undertaken to prepare it for analysis, including standardizing financial metrics across companies to enable direct comparisons. Normalization and data cleaning techniques were applied to ensure high data quality and reliability. According to Van den Broeck et al. (22), effective data cleaning involves detecting, diagnosing, and correcting abnormalities, which is essential for maintaining data integrity in financial analysis. Aguinis et al. (23) recommend best practices for handling outliers, emphasizing that proper outlier management is vital for accurate statistical results. Rousseeuw and Hubert (24) also highlight robust statistical methods for outlier detection, advocating for techniques that are less affected by anomalies, which improves data normalization reliability. Using these methods, outliers and missing data were addressed to enhance data quality. Z-score normalization was applied to revenue data to identify deviations from the mean, highlighting any potential outliers or anomalies in financial performance. This comprehensive data processing enables more accurate insights and supports better decision-making.
5. **Quality assurance**: Inspired by the frameworks of Redman (25) and Wang and Strong (26), a rigorous quality assurance process was implemented to ensure data integrity and usability. The process involved cross-referencing data with primary sources to identify and correct inconsistencies. Preliminary analyses were also conducted to validate the findings. Redman emphasizes the importance of strong quality control methods to uphold reliable data standards. Wang and Strong expand on this, viewing data quality as a multifaceted concept that includes accuracy, relevance, and user trust. Following these principles, our approach ensured that the data met comprehensive quality benchmarks.

## 5 Performance analysis

After dataset collection and processing, including anonymization and data normalization, we performed various analyses. The main objective is to evaluate the financial performance of selected companies in the public health and wellness sector. We aim to assess revenue trends, financial health ratios, and the impact of external shocks. Additionally, we benchmark these companies against industry averages. Overall, objective is to provide insights into market positioning and operational resilience in response to economic fluctuations.

**Figure 6:**
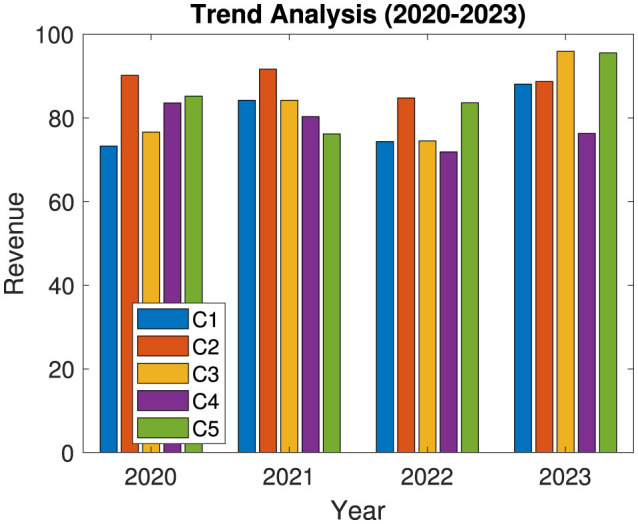
Revenue trend analysis of listed companies in the public health and wellness industry.

Figure 6 presents the annual revenue trends from 2020 to 2023 for five selected companies in the public health and wellness sector. Each company's revenue is depicted per year. This allows for a comparative view of growth patterns and fluctuations. Observing these trends aids in understanding the companies' market positioning, operational performance, and their ability to generate consistent revenue. Variations across years may indicate responses to industry-specific factors, market demand, or external economic conditions.

**Figure 7:**
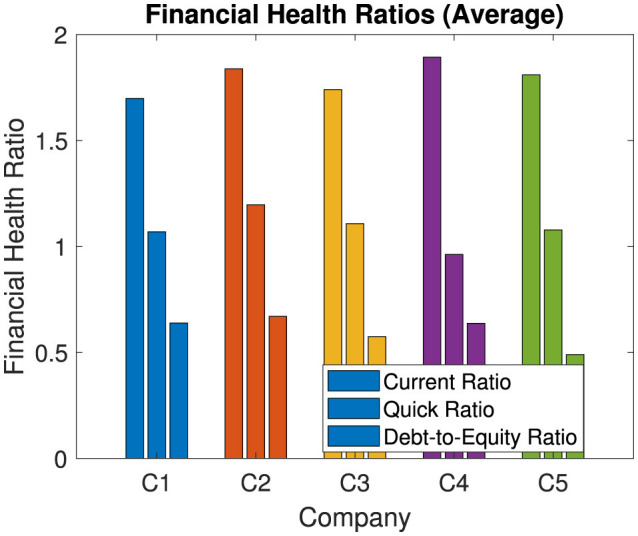
Financial health diagnosis of key ratios for selected public health and wellness companies (2020–2023 average).

Figure 7 illustrates the average financial health ratios for each selected company covering the current ratio, quick ratio, and debt-to-equity ratio. These ratios serve as key indicators of liquidity, financial stability, and risk exposure for each company. A higher current and quick ratio indicates greater ability to meet short-term obligations. The debt-to-equity ratio provides insight into the companies' financial control. These averages highlight each company's financial structure and can inform stakeholders of potential risks or financial strengths.

**Figure 8:**
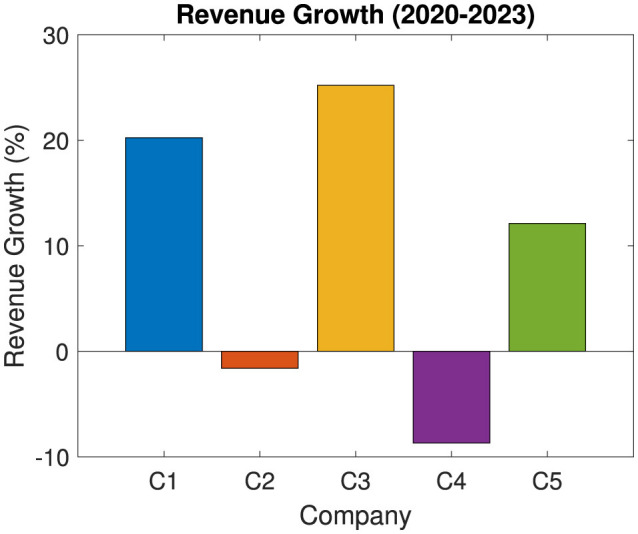
Impact of external shocks on revenue growth of listed companies in the public health and wellness sector.

Figure 8 examines the percentage growth in revenue. It highlights the impact of external market shocks, such as economic downturns or health crises, on financial performance. In this case study impact of COVID-19 is shown. The revenue growth calculates the percentage change in revenue from the initial to the final year as:


(1) $$\begin{array}{l} {\text{RevenueGrowth}}_{i}=\left(\frac{R_{i,\text{end}}-R_{i,\text{start}}}{R_{i,\text{start}}}\right)\times 100 \end{array}$$

where RevenueGrowth_*i*_ is the percentage growth in revenue for company *i* from the starting year to the ending year. *R*_*i*, end_ is the revenue of company *i* in the final year (2023). *R*_*i*, start_ is the revenue of company *i* in the initial year (2020).

Thus, it provides insight into each company's revenue growth over the specified period. Positive growth percentages suggest resilience and adaptability, whereas negative growth may indicate susceptibility to market disruptions. Overall, companies C1, C3, and C5, achieved positive growth, whereases C2 and C5 have shown negative growth. This analysis helps investors and stakeholders understand how external conditions have affected each company's financial stability over time. It also provides insight into strategic adjustments required to mitigate future risks.

**Figure 9:**
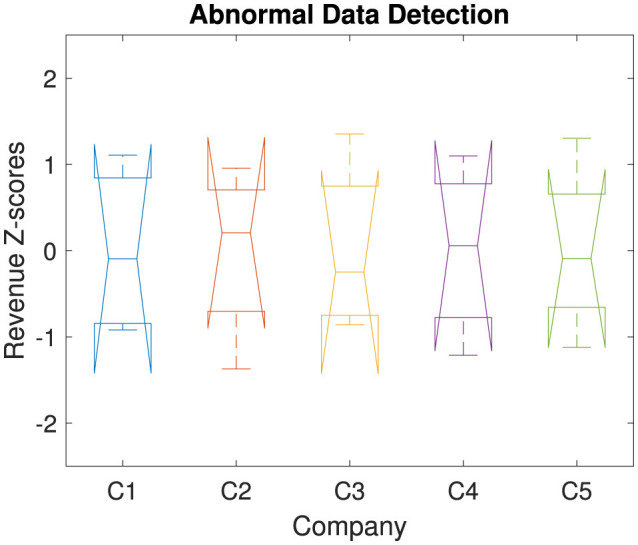
Detection of abnormal data using revenue Z-scores for public health and wellness companies.

Figure 9 visualizes the Z-scores of revenue data for each company across the analyzed years. It identifies any abnormal data points that may indicate potential outliers or anomalies. The Z-score normalization highlights deviations from mean revenue values, with significant deviations indicating abnormal patterns. The Z-score of revenue data can be computed as:


(2) $$\begin{array}{l} Z_{i,j}=\frac{R_{i,j}-\mu_{i}}{\sigma_{i}} \end{array}$$

where *Z*_*i, j*_ is the revenue Z-score for company *i* in year *j*. *R*_*i, j*_ represents the revenue of company *i* in year *j*. μ_*i*_ is the mean revenue of company *i* across all years. σ_*i*_ is the standard deviation of revenue for company *i* across all years.

**Figure 10:**
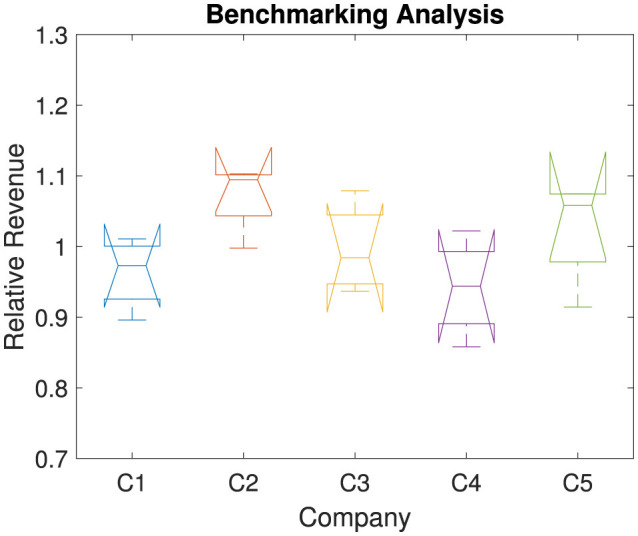
Benchmarking revenue performance against industry averages for selected companies.

High positive or negative *Z*_*i, j*_ values indicate abnormal revenue patterns, which are useful for identifying outliers and assessing financial stability. Detecting such anomalies is crucial for diagnosing potential operational and financial issues. These outliers could stem from atypical events, misreporting, or exceptional financial performance. This diagnostic tool enhances data integrity and enables accurate interpretation of company performance. In our study, we have found much variations among, i.e., abnormal patterns. But no outlier or extreme abnormality is found. Figure 10 compares each company's revenue performance as a ratio relative to the industry average across the years 2020 to 2023. The relative performance is shown per company, allowing for benchmarking against industry norms. It can be calculated as a ratio to the industry average. The industry average revenue for each year *j* is calculated by taking the mean revenue across all companies in that year as:


(3) $$\begin{array}{l} {\text{AR}}_{j}=\frac{1}{N}\overset{N}{\underset{i=1}{\sum }}R_{i,j} \end{array}$$

where *N* is the total number of companies. *R*_*i, j*_ represents the revenue of company *i* in year *j*.

The revenue performance for each company *i* in each year *j* is then computed as the ratio of the company's revenue to the industry average revenue for that year:


(4) $$\begin{array}{l} {\text{RP}}_{i,j}=\frac{R_{i,j}}{{\text{AR}}_{j}} \end{array}$$

Here, a revenue performance score >1 indicates that a company's revenue is above the industry average in that year. A score < 1 shows that the company's revenue is below the industry average for that year.

Therefore Figure 10 provides a clear view of industry positioning. This comparative analysis highlights which companies lead or lag relative to peers. Thus, this analysis offers strategic insights into competitive advantages and areas for potential improvement within the public health and wellness industry. Overall, in our study, we found that though we have seen negative growth in C2, but it achieves better revenue compared to all other companies (refer Figure 8). Similarly, though C3 shows significantly better growth, but in terms of revenue it is lacking compared to C2 and C5.

## 6 Recommendations and future scope

This study leads to various practical recommendations and highlight areas for further exploration. Implementing these recommendations and continue future research could enhance both individual company performance and broader industry stability.

### 6.1 Recommendations

1. **Financial liquidity and stability**: Companies should prioritize maintaining robust current and quick ratios to effectively meet short-term financial obligations. This focus is especially important due to the industry's susceptibility to market fluctuations and health crises. Maintaining healthy liquidity levels enables companies to respond to sudden disruptions more effectively and ensures continuity in operations.
2. **Debt management**: To balance growth opportunities with financial risk, firms should regularly review and manage their debt-to-equity ratios. Companies with high leverage are advised to explore alternative financing options, such as equity financing or strategic partnerships, to reduce financial strain and enhance long-term sustainability.
3. **Strategic planning**: Given the industry's sensitivity to external shocks, companies should adopt agile operational frameworks and flexible business models. This adaptability allows companies to improve quickly in response to market changes, regulatory updates, or consumer behavior shifts. It has already been demonstrated by companies that showed positive growth during the COVID-19 period.
4. **Financial monitoring and data integrity**: Companies should adopt advanced analytics as routine financial monitoring tools to identify any abnormal data points that could indicate potential operational or reporting issues. Early detection and intervention can prevent discrepancies from impacting financial reporting accuracy. This will improve stakeholder trust and compliance.
5. **Invest in consumer-centric solutions**: The public health and wellness sector is evolving rapidly. Investing in areas that align with emerging health trends and consumer demands can provide companies with a significant edge. Companies may consider enhancing digital health capabilities, expanding wellness service offerings, or integrating sustainability initiatives. These strategies offer potential paths to attract new consumer segments and drive growth.

### 6.2 Future scope

1. **Larger dataset**: Future research could extend this analysis to a larger dataset. This dataset would encompass more companies within the health and wellness industry. Such an expansion would allow for a more comprehensive view of industry trends, financial health patterns, and common challenges. Therefore, this could yield insights with broader applicability.
2. **External market factors**: Examining the specific impact of external market conditions on financial performance could help isolate the factors driving revenue fluctuations. These external market conditions include regulatory changes, economic policies, and health crises. This research can aid in developing risk mitigation strategies. These strategies would better prepare companies for external disruptions.
3. **Digital transformation impact**: Given the increasing importance of digital health, future research could explore how investment in digital tools and services impacts financial performance in the public health and wellness sector. Understanding this relationship would help companies prioritize digital strategies. It would also assist in identifying innovations that yield the highest returns.
4. **Environmental, social, and governance (ESG) metrics**: As stakeholders increasingly value corporate responsibility, it is important to study the relationship between Environmental, Social, and Governance (ESG) metrics and financial performance in this industry. This research could reveal how responsible practices align with revenue growth. It could also show the connection to consumer trust and investor interest.

## 7 Discussion and conclusion

The financial assessment and diagnosis of listed companies in the public health and wellness industry offer critical insights into the operational health, resilience, and financial stability of the sector. By examining revenue trends, financial health ratios, revenue growth, detection of abnormal data points, and benchmarking metrics, this analysis highlights both strengths and potential vulnerabilities of companies within this essential industry. These insights are invaluable to stakeholders and guide future decision-making and strategic planning.

The observed revenue trends from 2020 to 2023 reveal varied growth patterns, reflecting each company's unique market positioning, operational efficiency, and responsiveness to industry challenges. Companies that show consistent or rising revenue generally indicate strong market positioning and effective management practices, which are vital in an industry subject to fluctuations in consumer demand and regulatory conditions. In contrast, companies with stagnant or declining revenue may need to reevaluate their operational strategies and market approach. These trends emphasize the importance of adaptability and strategic foresight, as companies must continually align with changes in consumer behavior, regulatory policies, and economic factors to remain competitive.

Analysis of financial health ratios, such as the current ratio, the quick ratio, and the debt-to-equity ratio, provides additional information on the liquidity, solvency, and financial risk exposure of each company. High current and quick ratios are favorable, as they indicate an ability to meet short-term obligations, which is especially crucial in an industry where companies must remain prepared for sudden market shifts or health crises. The debt-to-equity ratio reveals each company's leverage level, with excessively high ratios suggesting over-reliance on debt that could pose risks in times of financial strain. These ratios collectively allow stakeholders to gauge individual company health, assess risk levels, and identify red flags that may require attention to prevent liquidity issues or excessive leverage from impacting long-term growth.

Revenue growth analysis demonstrates the effects of economic disruptions, health crises, and shifts in consumer demand on company performance. Calculating percentage revenue growth over the period allows stakeholders to assess each company's adaptability to market conditions. Companies showing positive growth amidst challenging times, such as the COVID-19 pandemic, likely possess robust crisis management practices, operational flexibility, or a strong market presence that allows them to thrive even in adversity. Those with negative growth may need to reassess their strategies to improve resilience. Recognizing and emulating best practices from adaptable and resilient companies can guide underperformers in addressing their weaknesses and aligning more closely with industry standards.

Detecting and diagnosing abnormal data points through statistical tools, such as Z-scores, is essential for ensuring data integrity, accuracy in financial reporting, and effective financial assessments. Significant deviations from the norm can suggest operational issues, data misreporting, or even fraud, which can mislead stakeholders and distort performance assessments. In this analysis, no extreme outliers or major abnormalities were identified, suggesting reliable revenue reporting across the sector. Ongoing monitoring for such deviations helps maintain financial transparency, which is critical in sustaining stakeholder trust and complying with regulatory standards.

Benchmarking against industry averages offers a framework for understanding relative competitiveness within the market. Calculating revenue performance as a ratio of each company's revenue to the industry average provides valuable insight into leading and lagging companies. Firms with above-average revenue performance demonstrate competitive advantages, whether through operational efficiency, brand strength, or market alignment, positioning them for sustained growth. Conversely, companies below the industry average may benefit from targeted interventions to improve their market positioning. Such benchmarking not only highlights areas for growth and improvement but also informs strategic decisions, investment opportunities, and potential collaborations or partnerships within the industry.

Overall, the financial assessment and diagnostic analysis illustrate the multifaceted nature of evaluating financial health and performance in the public health and wellness industry. Companies that perform well across key financial metrics and adapt effectively to changing market conditions establish themselves as industry leaders. For those struggling with issues such as negative revenue growth or high debt levels, this analysis provides actionable insights for strategic improvement. Additionally, maintaining financial transparency, ensuring data accuracy, and utilizing benchmarking are critical for fostering sustainable growth and resilience in this rapidly evolving industry.

Overall, this study presents the significance of sound financial practices, adaptability, and competitive awareness within the public health and wellness sector. The combination of quantitative financial metrics and strategic insights allows stakeholders to make informed decisions that enhance operational stability, drive growth, and foster resilience. For investors and policymakers, these findings provide a roadmap to identify promising investment opportunities, promote industry stability, and support continued progress in a sector that is integral to societal well-being. Through continuous monitoring, financial diligence, and strategic planning, companies in the public health and wellness industry can navigate market complexities and position themselves for future success.

## Data Availability

The original contributions presented in the study are included in the article/supplementary material, further inquiries can be directed to the corresponding author.

## References

[1] Wenhai Z Qiong Z Wei H Jun Y. The Xinhua News Agency. Xi Jinping sent a message of condolences to French President Emmanuel Macron on the COVID-19 outbreak in France[EB/OL]. Chinese Ment Health J. (2022) 36:626.

[2] Li CG Luo JW. Deeply understanding the logical path of “human health community”. Guangming Daily, 2022-04-21 (006).

[3] Fang D. Research on enterprise financial risk control in the “internet plus” era. Account Learn. (2020) 2:45–7. 10.12783/dtssehs/icesd2017/11682

[4] Ou GL Wu G Zhu XB. Research on financial risk warning of listed real estate enterprises based on factor analysis. Soc Scient. (2018) 09:56–63. 10.1061/9780784483237.085

[5] Hertzel MG Li Z Officer MS Rodgers KJ. Inter-firm linkages and the wealth effects of financial distress along the supply chain. J Financ Econ. (2008) 87:374–87. 10.1016/j.jfineco.2007.01.005

[6] Beaver WH. Financial ratio as predictors of failure. J Account Res. (1966) 4:77–111. 10.2307/2490171

[7] Westerfield RW Jordan BD. Fundamentals of Corporate Finance. Madrid: McGraw-Hill (1995). p. 264-286.

[8] Marjorie B. Predicting corporate financial distress: reflections on choice-based sample bias. J Econ Finance. (2002) 26:77–80. 10.1007/BF02755985

[9] Tian XR Jiang HD Liu JL. The research of financial risk warning model about real estate enterprises in China. Adv Mat Res. (2014) 989:2625–8. 10.4028/www.scientific.net/AMR.989-994.2625

[10] Xiang DW. On financial risk. Account Res. (1994) 04:21–5.

[11] Zhang JY Gao X. Causes and prevention strategies of financial risks in enterprises. Account Res. (2020) 4:166–7. 10.2308/acch-10264

[12] Tang H Jia Z. The causes and prevention strategies of financial risks in enterprises. Modern Bus. (2021) 3:190–2. 10.1056/NEJM198611273152231 3773975

[13] Zhang T Yu TF. Research on enterprise financial risk management and control. China Manage Inf. (2021) 2:18–9. 10.25236/busem.2017.02

[14] Shivam Azad SL Devi T Sustainable Sustainable development and investor confidence: the safe-haven appeal of green-bond issuing firms. Sustain Dev. (2024). 10.1002/sd.3172

[15] Liu M Wu H. Research on enterprise financial risk assessment based on big data. Manag Serv Sci. (2019) 3:62–6. 10.1109/ICITBS.2018.00142

[16] Zheng ZP. Financial risk assessment model based on principal component analysis. Jiangsu Commer Forum. (2018) 21:50–5. 10.1109/TBME.2006.876623 16916086

[17] Brigham EF Gapenski LC Ehrhardt MC. Financial Management; Theory and Practice (Book and diskette package). Los Angeles: Harcourt College Publishers. (1998).

[18] Koller T Goedhart M Wessels D. Valuation: Measuring and Managing the Value of Companies. New York: John Wiley & Sons. (2010).

[19] Gadotti A Rocher L Houssiau F Creu AM de Montjoye YA. Anonymization: The imperfect science of using data while preserving privacy. Sci Adv. (2024) 10:eadn7053. 10.1126/sciadv.adn7053 39018389 PMC466941

[20] Xu H Zhang N. Implications of data anonymization on the statistical evidence of disparity. Manage Sci. (2022) 68:2600–18. 10.1287/mnsc.2021.402819642375

[21] El Emam K Dankar FK. Protecting privacy using k-anonymity. J Am Med Inform Assoc. (2008) 15:627–37. 10.1197/jamia.M2716 18579830 PMC2528029

[22] Van den Broeck J Argeseanu Cunningham S Eeckels R Herbst K. Data cleaning: detecting, diagnosing, and editing data abnormalities. PLoS Med. (2005) 2:e267. 10.1371/journal.pmed.0020267 16138788 PMC1198040

[23] Aguinis H Gottfredson RK Joo H. Best-practice recommendations for defining, identifying, and handling outliers. Organ Res Methods. (2013) 16:270–301. 10.1177/1094428112470848

[24] Rousseeuw PJ Hubert M. Robust statistics for outlier detection. Data Mining Knowl Disc. (2011) 1:73–9. 10.1002/widm.2

[25] Redman TC. Data Quality: The Field Guide. Springfield: Digital Press. (2001).

[26] Wang RY Strong DM. Beyond accuracy: what data quality means to data consumers. J Manag Inf Syst. (1996) 12:5–33. 10.1080/07421222.1996.11518099

